# Integrative Network Analysis and Machine Learning Identify Cell Adhesion‐Related Biomarkers and Immune Heterogeneity in Allergic Rhinitis

**DOI:** 10.1002/iid3.70562

**Published:** 2026-10-19

**Authors:** Guoshun He, Liying Zhang

**Affiliations:** ^1^ Department of Otorhinolaryngology Yiwu Traditional Chinese Medicine Hospital Yiwu China; ^2^ Beiyuan Subdistrict Community Health Service Center Yiwu China

**Keywords:** allergic rhinitis, cell adhesion molecules, immune infiltration, machine learning, molecular subtype

## Abstract

**Background:**

Allergic rhinitis (AR) represents a highly prevalent inflammatory disorder of the nasal mucosa, but molecular indicators capable of capturing its disease‐specific transcriptional features remain insufficiently defined. This study aimed to identify cell adhesion‐related diagnostic biomarkers for AR and to investigate their immune relevance and molecular heterogeneity.

**Methods:**

Publicly available AR microarray datasets were analyzed, with GSE75011 serving as the discovery dataset and GSE46171 and GSE40037 used to test the reproducibility of the diagnostic model. A predefined adhesion‐related gene set was curated from MSigDB to provide the biological search space for biomarker screening. Genes showing disease‐associated expression changes were overlapped with this adhesion gene set to obtain AR‐associated adhesion genes. Their biological functions and interaction structure were examined through enrichment analysis, protein interaction mapping, and module extraction. Diagnostic candidates were prioritized by integrating three complementary feature‐selection strategies: least absolute shrinkage and selection operator regression, random forest modeling, and support vector machine‐recursive feature elimination. The selected markers were then assessed for diagnostic discrimination, individualized prediction, calibration performance, and potential clinical net benefit. In addition, immune infiltration, upstream regulatory networks, candidate compounds, and non‐negative matrix factorization‐based molecular subtypes were explored.

**Results:**

A total of 38 differentially expressed adhesion‐related genes were identified in AR, and 13 hub genes were extracted from the key protein–protein interaction module. Integrated machine learning ultimately prioritized TNFSF14 and CD83 as diagnostic biomarkers. The combined TNFSF14/CD83 model showed strong diagnostic separation in GSE75011, with an area under the curve (AUC) of 0.968, and maintained external performance in GSE46171 and GSE40037, with AUC values of 1.000 and 0.758, respectively. Both biomarkers were linked to altered immune‐cell features, particularly T‐cell‐related signatures. Using the two‐marker expression pattern, AR samples could be separated into two exploratory molecular patterns that showed differences in immune profiles and enriched biological processes.

**Conclusions:**

TNFSF14 and CD83 may represent promising candidate diagnostic biomarkers associated with AR‐related immune alterations. These findings also reveal immune heterogeneity within AR and provide a bioinformatics‐based framework for biomarker discovery and molecular stratification.

## Summary

- TNFSF14 and CD83 were identified as cell adhesion‐related diagnostic biomarkers for allergic rhinitis.
- A two‐gene model showed favorable diagnostic performance across the training and external validation cohorts.
- Allergic rhinitis exhibited immune heterogeneity and could be stratified into two molecular subtypes.

## 1 Introduction

Allergic rhinitis (AR) develops when allergen exposure triggers IgE‐dependent immune activation in the nasal mucosa, leading to recurrent upper‐airway inflammation and typical nasal symptoms [1]. It affects hundreds of millions worldwide and its prevalence has been rising in recent decades [2]. AR often begins in childhood or adolescence and frequently coexists with asthma and other atopic diseases [3]. Clinically, patients suffer from nasal congestion, sneezing, rhinorrhea and itching, which can persist long after allergen exposure and lead to significant impairment in quality of life and productivity [4, 5]. Epidemiological studies estimate that roughly 10%–30% of the population has AR, with rates even higher in urban or industrialized regions [6, 7]. The pathophysiology of AR involves a biphasic immune response: on allergen challenge, mast cells release histamine and leukotrienes causing immediate symptoms, while a later cascade of T helper type 2 (Th2) cytokines (especially IL‐4 and IL‐13) drives recruitment of eosinophils, basophils and lymphocytes into the nasal tissue [8]. This Th2‐dominated inflammation is central to AR, as excessive IL‐4/IL‐13 production perpetuates nasal mucosal inflammation and barrier dysfunction. Overall, AR is a systemic inflammatory disease with a heavy clinical and socioeconomic burden, yet it remains incurable and often difficult to diagnose precisely [9].

At the cellular level, leukocyte recruitment to the airway depends critically on adhesion molecules (CAMs) expressed by endothelial and epithelial cells [10]. During allergic airway inflammation, cytokine stimulation activates endothelial and epithelial adhesion programs, including ICAM‐1, VCAM‐1, and selectin‐related pathways, thereby facilitating leukocyte attachment and transmigration into the nasal mucosa [11]. These CAMs bind circulating eosinophils and T cells, guiding their exit from blood into the nasal mucosa. Indeed, studies of AR patients show markedly increased ICAM‐1 and VCAM‐1 expression in nasal tissue compared to healthy controls [10]. Functionally, blocking CAM interactions reduces airway inflammation in animal models, underscoring their importance: for example, VCAM‐1–VLA‐4 blockade prevents antigen‐induced eosinophil infiltration in mouse airways [12]. In short, adhesion molecules act as gatekeepers of allergic inflammation. By mediating eosinophil and lymphocyte trafficking, CAMs are now recognized as central players in AR pathogenesis [13]. Consistent with this, AR patients exhibit elevated systemic and local inflammatory markers, including CRP, PCT, IL‐1β, and TNF‐α, which correlate with disease severity and nasal functional impairment [14].

Here, we focused on adhesion‐associated immune transcriptional alterations in AR to identify candidate diagnostic markers. Rather than restricting the analysis to classical structural adhesion molecules, this strategy aimed to capture broader immune regulatory programs involved in cell‐cell interaction and inflammatory communication. This workflow prioritized TNFSF14 and CD83 as a two‐gene diagnostic panel that distinguished AR samples from controls across the discovery and validation datasets. Further analyses indicated that these biomarkers were closely associated with immune‐related pathways and altered T‐cell features in AR. Their expression profiles further separated AR cases into two biologically divergent groups with different immune‐context features. Together, these findings provide new insight into the molecular heterogeneity of AR and suggest TNFSF14 and CD83 as promising diagnostic biomarkers.

## 2 Methods

### 2.1 Data Acquisition

Public AR‐related microarray expression profiles were collected from the Gene Expression Omnibus (GEO) repository (https://www.ncbi.nlm.nih.gov/geo/). Expression matrices were obtained from the original GEO processed datasets. Each dataset was analyzed independently according to its original preprocessing pipeline, and datasets were not merged across platforms to avoid potential batch effects. GSE75011 was selected as the discovery cohort, containing 15 AR samples and 25 healthy controls. In addition, two independent external validation cohorts, GSE46171 and GSE40037, were obtained from the same database. GSE46171 included 6 AR and 3 control samples, while GSE40037 included 10 AR and 12 control samples. For adhesion‐focused screening, we used the GOBP_CELL_ADHESION gene set (M45069) from MSigDB and retained 1542 adhesion‐related genes (ARGs) for subsequent analyses (Supporting Information S1: Table S1).

### 2.2 Identification of Differentially Expressed Cell Adhesion‐Related Genes

The expression matrix of GSE75011 was analyzed with limma to screen transcripts that differed between AR and healthy control samples. Genes meeting the criteria of *p* < 0.05 and |log_2_FC | > 0.585 were considered differentially expressed. This relatively inclusive threshold was applied during the initial screening stage to avoid missing potentially relevant adhesion‐associated candidates, followed by subsequent PPI network analysis and multiple machine‐learning approaches for further prioritization. Genes showing AR‐associated expression changes were overlapped with the adhesion‐related gene list, and the shared genes were defined as differentially expressed adhesion‐related genes (DEARGs). To characterize the biological context of the DEARGs, Gene Ontology (GO) functional terms and Kyoto Encyclopedia of Genes and Genomes (KEGG) pathways were analyzed through clusterProfiler.

### 2.3 Protein Interaction Mapping and Hub‐Module Extraction

Potential protein‐level connections among the intersecting genes were queried in STRING, and interactions with confidence scores ≥ 0.15 were retained for network construction. The network was visualized in Cytoscape (version 3.8.0; https://cytoscape.org/). Dense gene clusters within the interaction network were extracted with the MCODE plug‐in using degree cutoff 2, node score cutoff 0.2, K‐core 2, and max depth 100. MCODE returned two network modules, and the module with the top score was carried forward as the core interaction module. Genes located in this core module were retained as hub candidates for subsequent screening. For these hub candidates, expression differences between AR and control samples were assessed, and their biological functions were further characterized by GO enrichment analysis in clusterProfiler.

### 2.4 Machine Learning‐Based Identification of Key Diagnostic Features

To narrow the hub‐gene list to markers with stronger diagnostic relevance, we introduced three complementary machine‐learning screening approaches. Random forest (RF), least absolute shrinkage and selection operator (LASSO), and support vector machine‐recursive feature elimination (SVM‐RFE) were implemented in R through the randomForest, glmnet, and e1071 packages, respectively, to rank and filter candidate diagnostic features from different algorithmic perspectives. The genes retained by the three algorithms were then intersected, and overlapping genes were defined as machine learning‐derived candidate features for subsequent diagnostic evaluation.

### 2.5 Diagnostic Performance Evaluation and Model Validation

In GSE75011, the discriminatory performance of the machine‐learning‐selected genes was quantified by receiver operating characteristic (ROC) analysis implemented with the pROC package. Genes were retained for biomarker evaluation when their Area Under the Curve (AUC) exceeded 0.70, indicating acceptable separation between AR and control samples. These retained genes were then combined to generate a diagnostic classifier. For sample‐level prediction, the selected markers were incorporated into a nomogram with the rmda package, and calibration analysis was used to examine the agreement between predicted and observed diagnostic probabilities. The potential usefulness of the classifier across different threshold probabilities was further examined by decision curve analysis (DCA) through rmda. The reproducibility of the diagnostic classifier was tested in two independent GEO datasets, GSE46171 and GSE40037.

### 2.6 Expression Assessment and Gene‐Specific Functional Exploration of Diagnostic Markers

The transcript levels of the selected diagnostic markers were compared between AR samples and healthy controls. To explore the functional background linked to each marker, samples were ranked according to individual biomarker expression, and enrichment analysis was performed with the c5.go.bp.v2024.1.Hs.symbols.gmt gene set.

### 2.7 Immune Landscape Analysis

Immune‐cell‐related signature scores were calculated for AR and control samples using single‐sample gene set enrichment analysis (ssGSEA) to characterize disease‐associated immune variation. CIBERSORT was additionally applied to estimate immune‐cell fractions from another computational perspective. The associations between diagnostic‐marker expression and immune‐cell estimates were further quantified by Pearson correlation analysis.

### 2.8 Upstream Regulatory Mapping and Candidate Compound Screening

Potential transcriptional regulators of the diagnostic markers were queried through NetworkAnalyst (https://www.networkanalyst.ca/), and the predicted transcription factor (TF)–mRNA pairs were organized into a regulatory network. Experimentally supported miRNA interactions targeting the diagnostic genes were then obtained from miRTarBase (https://mirtarbase.cuhk.edu.cn/) to define candidate post‐transcriptional regulators. The miRNA–mRNA links were combined with corresponding lncRNA–miRNA relationships to assemble a putative competing endogenous RNA (ceRNA) network. The resulting regulatory networks were visualized in Cytoscape. For compound screening, the diagnostic genes were analyzed through Enrichr (https://maayanlab.cloud/modEnrichr/), and DSigDB‐based (http://dsigdb.tanlab.org/DSigDBv1.0/) enrichment was used to prioritize small molecules potentially associated with these markers.

### 2.9 Diagnostic Marker‐Based Stratification of AR Molecular Patterns

To assess whether AR samples could be separated into molecularly different groups, non‐negative matrix factorization (NMF) clustering was applied to the training cohort using the expression profiles of the selected diagnostic markers. The subtype distribution was further examined by principal component analysis (PCA) to visualize whether the NMF‐defined groups occupied different transcriptomic spaces. Boxplots were generated to compare diagnostic‐marker expression between the NMF‐derived subtypes. Subtype‐related immune variation was evaluated by ssGSEA, and immune‐cell composition was further profiled using CIBERSORT and MCP‐counter. Genes differing between the two subtypes were screened with cutoffs of *p* < 0.05 and |log_2_FC | > 0.585, followed by GO enrichment analysis of the subtype‐associated differentially expressed genes (DEGs) in clusterProfiler.

## 3 Results

### 3.1 DEARGs in AR

We first compared AR samples with healthy controls in GSE75011 to screen for disease‐associated transcriptional changes related to cell adhesion. The volcano plot summarizes the genome‐wide expression differences between the two groups (Figure 1A). By intersecting the DEGs with the predefined cell adhesion‐related gene set, a total of 38 DEARGs were obtained (Figure 1B). We next examined the functional background of these DEARGs to clarify their potential biological involvement in AR. GO annotation linked these genes to adhesion‐ and immunity‐related processes, including positive regulation of cell‐cell adhesion, leukocyte cell‐cell adhesion regulation, cell activation, cell‐substrate junctions, focal adhesion, and cytokine receptor binding (Figure 1C). KEGG pathway analysis further showed enrichment in several inflammation‐ and adhesion‐related pathways, such as IgSF CAM signaling, cytokine‐cytokine receptor interaction, and cell adhesion molecule (CAM) interaction (Figure 1D). The expression landscape of the 38 DEARGs across AR and healthy control samples is illustrated in the heatmap, which revealed a distinct transcriptional pattern between the two groups (Figure 1E). Potential protein‐level connections among the 38 DEARGs were then mapped through STRING and displayed in Cytoscape (Figure 1F). MCODE detected two densely connected modules within the protein–protein interaction (PPI) network, and the module with the highest score was selected for further analysis (Figure 1G). The top‐scoring module included 13 genes, which were carried forward as hub candidates. Detailed information on the MCODE‐derived modules, including module scores and member genes, is provided in Supporting Information S1: Table S2. Functional annotation of the 13 hub candidates mainly pointed to immune‐regulatory processes, such as regulation of CD4‐positive alpha‐beta T‐cell differentiation, T‐cell activation, cell‐cell adhesion, and leukocyte cell‐cell adhesion (Figure 1H). Expression comparison confirmed significant AR‐control differences for all 13 hub candidates (Figure 1I). Among them, CX3CR1 and CADM1 were upregulated in AR samples, whereas NR4A3, PDCD1, MAP3K8, TNFSF14, ZC3H12A, DUSP1, NLRP3, SMAD7, TBX21, CD83, and ICOSLG were downregulated.

**Figure 1:**
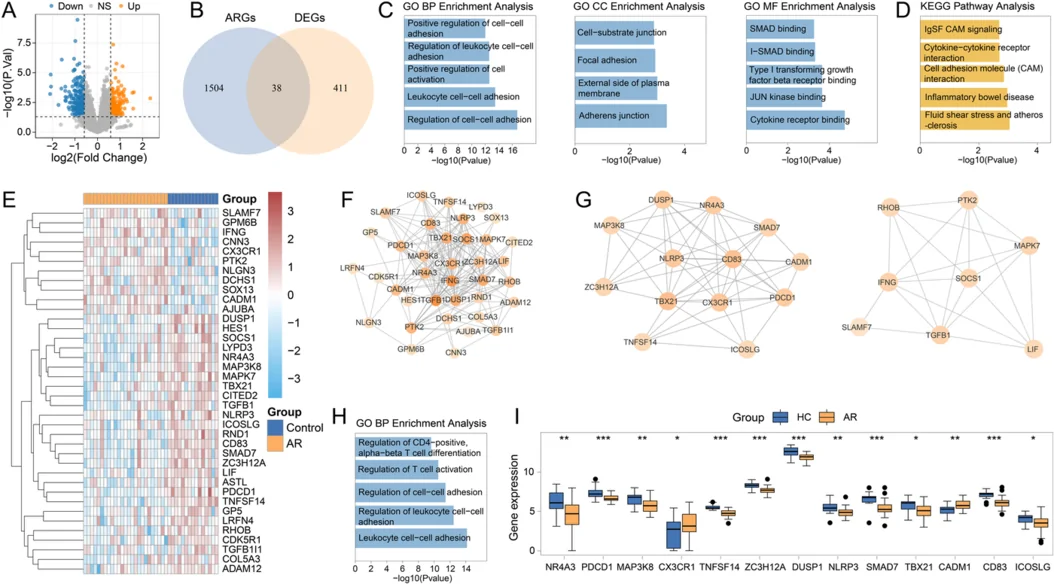
Identification and characterization of DEARGs in AR. (A) Volcano plot summarizing AR‐control transcriptional differences in GSE75011. (B) Venn diagram identifying genes shared by DEGs and ARGs. (C) GO annotation of the 38 DEARGs across biological process (BP), cellular component (CC), and molecular function (MF) categories. (D) KEGG pathway enrichment analysis of the DEARGs. (E) Heatmap of the 38 DEARGs across AR and control samples. (F) PPI network constructed from the 38 DEARGs. (G) Two MCODE‐derived PPI modules, with the top‐scoring module shown on the left and the second module shown on the right. (H) GO biological process annotation for the 13 hub candidates from the core module. (I) Expression comparison of the 13 hub candidates between healthy controls and AR samples. *p < 0.05, **p < 0.01, ***p < 0.001.

### 3.2 Cross‐Algorithm Screening Nominated TNFSF14 and CD83 as AR Diagnostic Markers

The 13 hub candidates were next filtered through three parallel algorithms to focus on features with stronger diagnostic relevance. In the LASSO procedure, cross‐validation selected the lambda.min setting, under which five genes remained in the model (Figure 2A,B). The RF model ranked six genes above the MeanDecreaseGini threshold of 1, indicating higher variable importance (Figure 2C,D). In addition, SVM‐RFE showed the lowest classification error when three features were included, and therefore three genes were selected from this model (Figure 2E). The shared output of LASSO, RF, and SVM‐RFE consisted of two genes, TNFSF14 and CD83, which were retained as the final diagnostic candidates (Figure 2F). Their ability to distinguish AR from controls was evaluated in GSE75011 using ROC curves. In this cohort, TNFSF14 and CD83 yielded AUC values of 0.931 and 0.845, respectively, supporting their individual diagnostic value (Figure 2G). A two‐gene diagnostic model was then established, and its performance exceeded that of either single marker in the training set, reaching an AUC of 0.968 (Figure 2H). When applied to the validation datasets, the two‐gene classifier reached an AUC of 1.000 in GSE46171 and 0.758 in GSE40037, indicating that the classifier retained discrimination across independent datasets, although the extremely small sample size of GSE46171 warrants cautious interpretation (Figure 2I,J). At the single‐gene level, CD83 retained moderate diagnostic value in both validation cohorts, while the performance of TNFSF14 varied substantially across datasets. Across the three cohorts, the expression patterns of TNFSF14 and CD83 were not completely identical, but both genes tended to be reduced in AR samples overall (Figure 2K). In the training cohort GSE75011, both TNFSF14 and CD83 were significantly downregulated in AR. In GSE46171, TNFSF14 was still significantly lower in AR, while the difference in CD83 expression was not statistically significant. In GSE40037, CD83 was significantly reduced in AR samples, while TNFSF14 showed no significant difference between groups. TNFSF14 and CD83 were then incorporated into a nomogram for sample‐level AR probability estimation (Figure 2L). Calibration analysis showed that the predicted probabilities were generally consistent with the observed diagnostic status (Figure 2M). DCA further suggested potential clinical usefulness of the two‐gene classifier across multiple threshold settings (Figure 2N).

**Figure 2:**
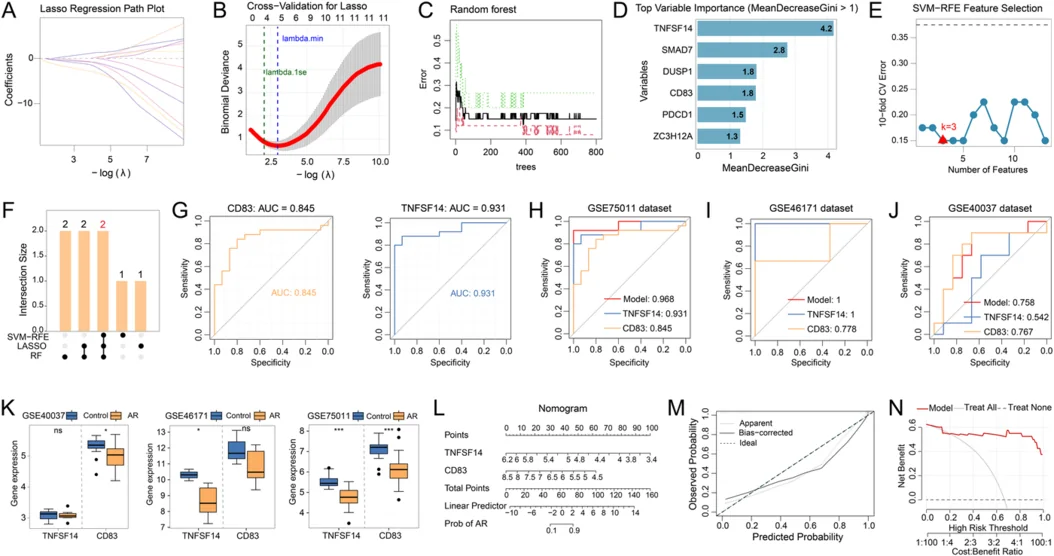
Selection and validation of the TNFSF14/CD83 diagnostic classifier in AR. (A) LASSO coefficient trajectories for the 13 hub candidates. (B) Cross‐validation curve used to determine the optimal LASSO penalty parameter. (C) RF error rate plot. (D) RF‐ranked genes with MeanDecreaseGini values above 1. (E) SVM‐RFE curve identifying the feature number associated with the minimum classification error. (F) Shared genes retained by LASSO, RF, and SVM‐RFE. (G) ROC curves for TNFSF14 and CD83 in GSE75011. (H) ROC curve of the TNFSF14/CD83 classifier in GSE75011. (I) Validation performance of the classifier in GSE46171. (J) Validation performance of the classifier in GSE40037. (K) Boxplots showing the expression levels of TNFSF14 and CD83 in control and AR samples across three datasets (from left to right: GSE40037, GSE46171, and GSE75011). (L) TNFSF14/CD83‐based nomogram for estimating AR probability. (M) Calibration curve of the nomogram. (N) DCA of the diagnostic model.

### 3.3 Functional and Regulatory Characterization of TNFSF14 and CD83

We next examined the biological contexts associated with CD83 and TNFSF14 by analyzing enrichment patterns linked to each marker. The enrichment profiles suggested that CD83 was mainly associated with biological processes such as negative regulation of RNA biosynthetic process, negative regulation of transcription by RNA polymerase II, and response to oxygen levels. In contrast, TNFSF14 was more closely linked to cell activation, intracellular signaling cassette, and nervous system process‐related signatures (Figure 3A). Potential transcriptional regulators of CD83 and TNFSF14 were queried through NetworkAnalyst, and the predicted TF–mRNA pairs were organized into regulatory networks (Figure 3B). Both biomarkers were connected to multiple candidate TFs, whereas TNFSF14 exhibited a broader TF interaction profile than CD83. A putative ceRNA regulatory network centered on CD83 and TNFSF14 was then established to further characterize their upstream post‐transcriptional regulatory context (Figure 3C). Notably, TNFSF14 occupied a more central position within the network and was linked to a larger number of predicted non‐coding RNA regulators, suggesting that it may be subject to a more complex regulatory architecture than CD83. Candidate compounds associated with TNFSF14 and CD83 expression signatures were further explored through DSigDB‐based enrichment analysis. These predicted associations were considered exploratory, and the highest‐ranked compounds are summarized in Table 1, with the full output provided in Supporting Information S1: Table S3.

**Figure 3:**
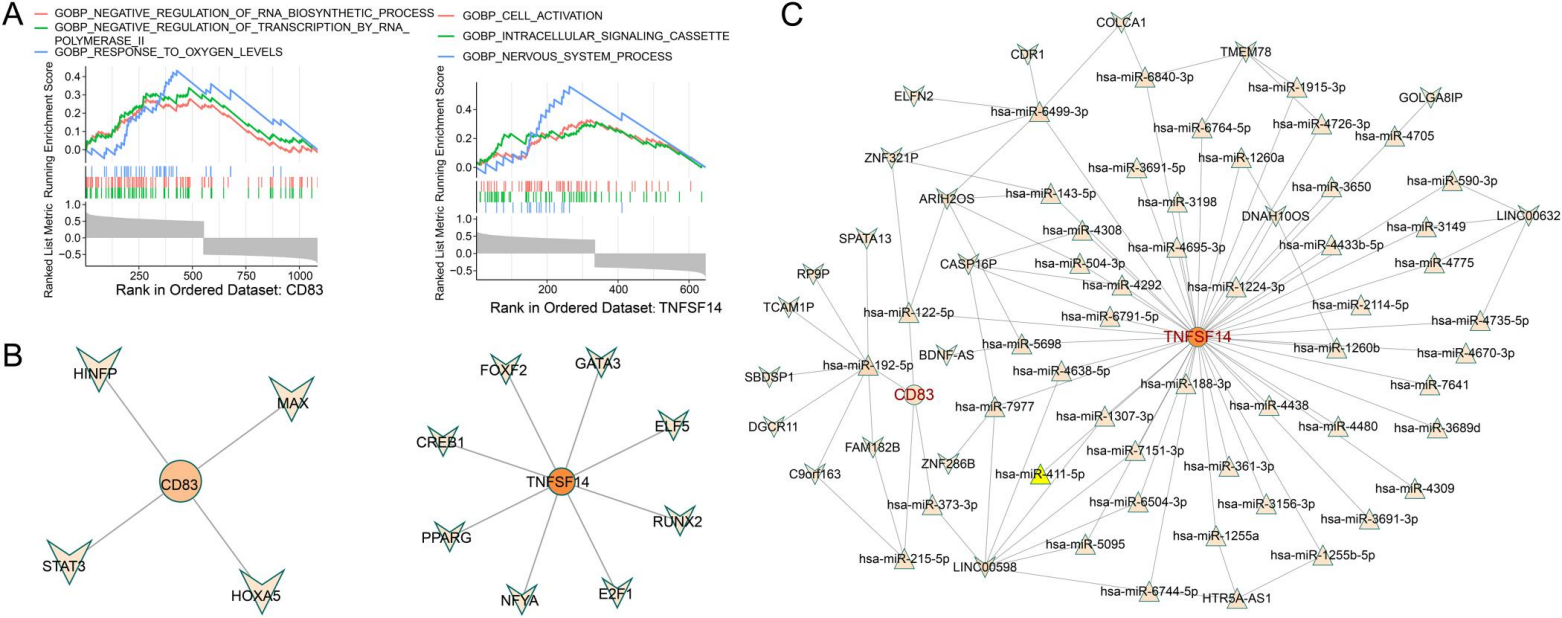
Functional annotation and regulatory mapping of TNFSF14 and CD83. (A) Gene‐specific enrichment profiles for CD83 and TNFSF14, with CD83‐related terms shown on the left and TNFSF14‐related terms shown on the right. (B) Predicted TF–mRNA regulatory networks of CD83 and TNFSF14. (C) Predicted ceRNA network involving CD83 and TNFSF14.

**Table 1:** Predicted compounds associated with TNFSF14 and CD83 expression signatures.

Term	*p* value	ARjusted *p* value	Odds ratio	Combined Score	Genes
puromycin HL60 UP	1.870E‐4	0.012	39452	338670.184	CD83; TNFSF14
menadione HL60 UP	2.781E‐4	0.012	39332	322037.578	CD83; TNFSF14
sanguinarine HL60 UP	3.677E‐4	0.012	39232	310257.538	CD83; TNFSF14
BETAMETHASONE VALERATE CTD 00005505	0.001	0.027	1998.8	13617.259	CD83

### 3.4 Immune Profiling Indicated T‐Cell‐Related Remodeling in AR

We next used ssGSEA to evaluate whether AR samples showed disease‐related shifts in immune‐cell signatures and immune functional states. At the immune‐cell level, AR samples showed lower activated dendritic cells (aDCs) and T follicular helper cells (Tfh) scores, while the T helper cell signature was moderately increased (Figure 4A). For immune functional signatures, most scores were similar between AR and control samples; however, T‐cell co‐stimulation was reduced in the AR group (Figure 4B). CIBERSORT was then used as an additional deconvolution method to examine immune‐cell composition in AR and control samples. CIBERSORT highlighted two altered CD4 T‐cell subsets: resting memory CD4 T cells were lower in AR, while naive CD4 T cells were higher (Figure 4C). These findings support the presence of a reshaped T‐cell compartment in the AR immune microenvironment. We further calculated Pearson correlations to determine how TNFSF14 and CD83 expression related to the inferred immune features. The correlation heatmap showed that CD83 and TNFSF14 exhibited distinct yet overlapping relationships with multiple immune‐cell subsets (Figure 4D), suggesting that both biomarkers may be closely involved in immune remodeling in AR rather than acting as isolated transcriptional signals.

**Figure 4:**
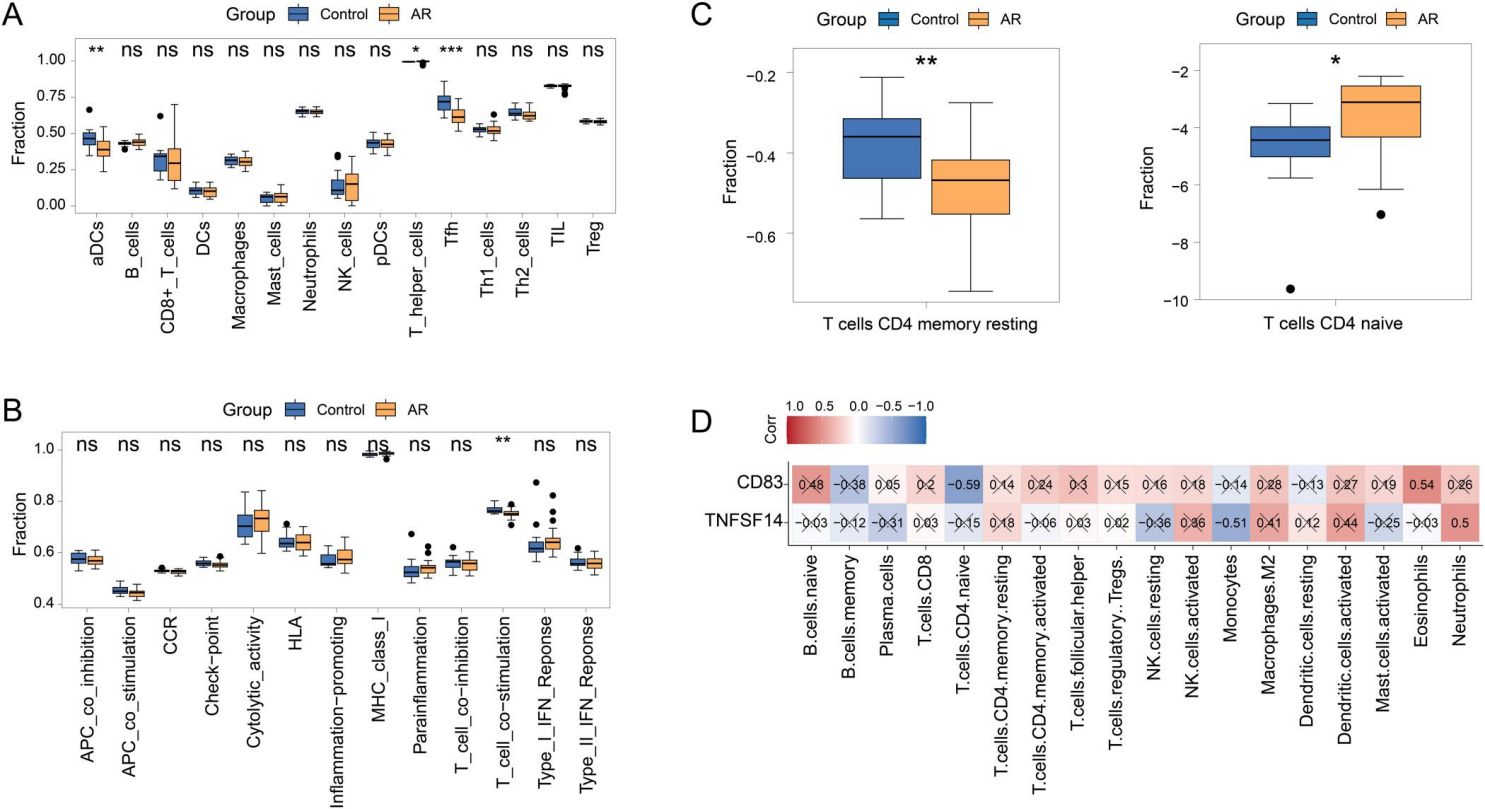
Immune infiltration analysis in AR. (A) ssGSEA‐derived immune‐cell signature scores in control and AR samples. (B) Immune functional scores estimated by ssGSEA in the two groups. (C) CIBERSORT‐inferred immune fractions highlighting altered resting memory CD4 T cells and naive CD4 T cells. (D) Correlation heatmap linking CD83 and TNFSF14 expression with immune‐feature estimates.

### 3.5 Exploratory Stratification Based on Diagnostic Markers Revealed Immune Heterogeneity in AR

To further investigate intradisease heterogeneity in AR, AR samples from the training cohort were stratified by NMF based on the expression profiles of the two diagnostic biomarkers. The NMF consensus results suggested a two‐group structure among AR samples (Figure 5A,B). PCA showed that the two NMF‐defined groups occupied different transcriptomic spaces, suggesting potential molecular differences between these exploratory groups (Figure 5C). The two diagnostic genes displayed different expression distributions across the NMF‐defined subtypes. TNFSF14 showed higher expression in Subtype 2, while CD83 was increased in Subtype 1 (Figure 5D), indicating that the two markers may reflect different molecular aspects of AR. We then examined whether this two‐subtype structure was accompanied by immune‐context differences. ssGSEA results showed subtype‐associated differences in several immune‐cell signatures, with notable changes involving pDCs, T helper cells, and tumor‐infiltrating lymphocyte (TIL)‐related scores (Figure 5E). In parallel, subtype‐associated divergence was also observed in selected immune functional signatures, including APC co‐stimulation, checkpoint activity, cytolytic activity, and T‐cell co‐stimulation (Figure 5F). MCP‐counter provided an additional overview of the immune‐cell composition across the two subtype groups and suggested differences in the relative abundance landscape of multiple immune cell populations (Figure 5G). To define the transcriptional programs separating the two subtypes, genes differing between Subtype 1 and Subtype 2 were screened and then analyzed for GO biological process enrichment. The subtype‐related DEGs were enriched for depyrimidination, ketone catabolism, and small‐molecule catabolic processes (Figure 5H), suggesting that metabolic‐related transcriptional differences may contribute to the observed separation, although these findings require further validation.

**Figure 5:**
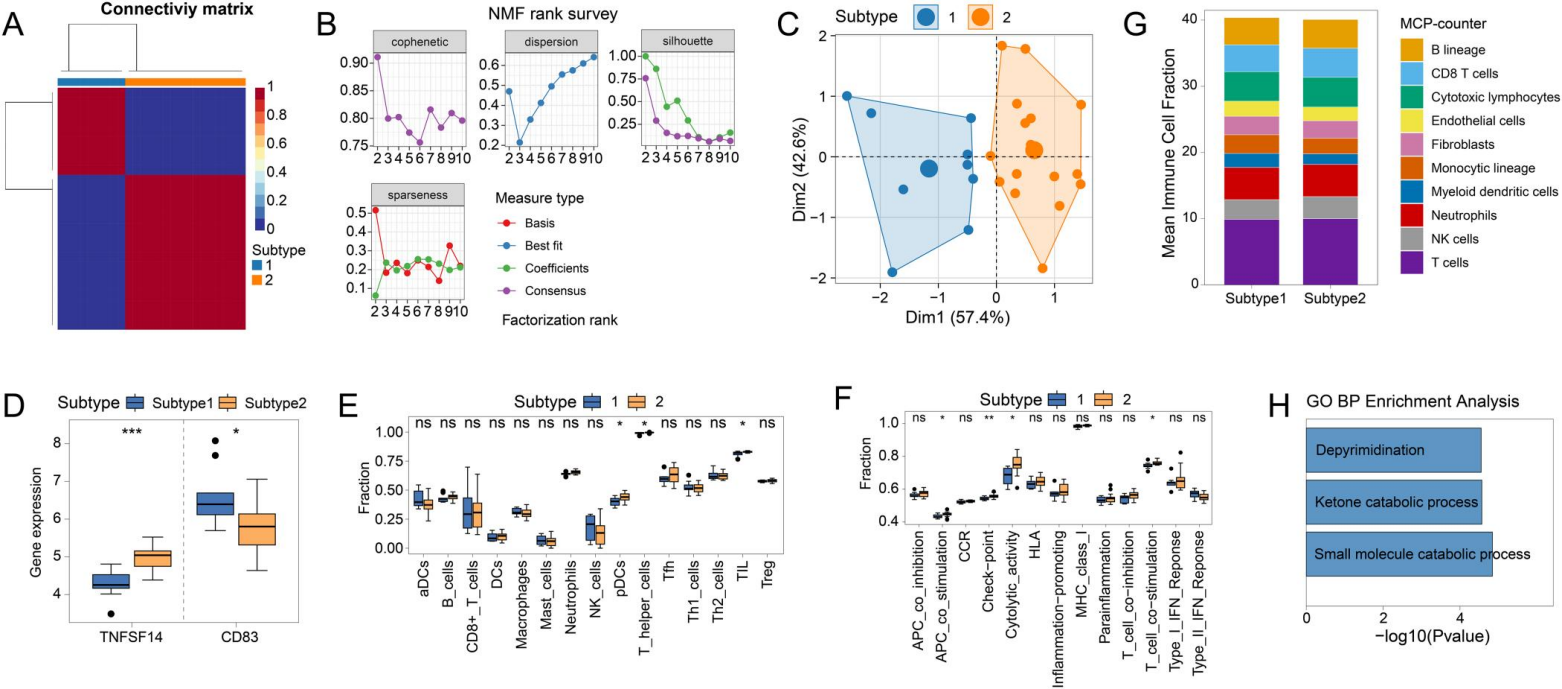
Molecular stratification of AR using TNFSF14 and CD83 expression. (A) NMF consensus matrix supporting a two‐subtype structure in AR samples. (B) Rank survey for selecting the optimal NMF cluster number. (C) PCA visualization of the two NMF‐defined AR subtypes. (D) TNFSF14 and CD83 expression distributions across the two subtypes. (E) Immune‐cell signature scores estimated by ssGSEA in the two subtypes. (F) ssGSEA‐derived immune functional scores across the two subtypes. (G) MCP‐counter‐based overview of immune‐cell composition across the two AR subtypes. (H) GO biological process terms enriched among subtype‐associated DEGs.

## 4 Discussion

In this study, 38 DEARGs were identified in AR versus controls. Functional enrichment analysis implicated these DEARGs in immune and cell‐cell adhesion processes, including leukocyte adhesion, T‐cell activation, and regulation of inflammatory effector responses. These pathways align with known AR biology, where endothelial and immune cell adhesion drives tissue inflammation. For instance, we found upregulation of CX3CR1 and CADM1, and downregulation of genes like TNFSF14, CD83, PDCD1, and others, consistent with dysregulated leukocyte signaling in AR. A PPI network highlighted 13 hub DEARGs, reflecting a tightly connected module of immune‐regulatory molecules.

Using three machine learning methods on the 13 hub genes, TNFSF14 and CD83 emerged as the top diagnostic candidates. Although TNFSF14 and CD83 are not classical adhesion molecules, they were identified from an adhesion‐associated immune transcriptional landscape. The adhesion‐focused gene set was therefore used as a biologically informed framework to capture immune interaction‐related alterations rather than exclusively structural adhesion proteins. TNFSF14 encodes a TNF superfamily ligand that acts as a costimulatory factor for lymphocyte activation [15]. It binds the herpesvirus entry mediator (HVEM) and lymphotoxin β receptors, stimulating T cell proliferation and modulating immune responses [16]. TNFSF14 has been implicated in allergic airway diseases: for example, studies in asthma models have shown that LIGHT promotes Th2‐driven inflammation and airway remodeling [17]. In our data, TNFSF14 was significantly downregulated in AR patients compared to controls. This inverse expression pattern is noteworthy because TNFSF14 has been implicated in immune activation in other inflammatory contexts. However, the decreased TNFSF14 expression observed in AR should not be interpreted as direct evidence of impaired LIGHT signaling or a causal role in disease development [18]. Possible explanations include altered immune‐cell composition, disease‐stage‐dependent regulation, or compensatory immune responses. Further functional studies are required to clarify the biological significance of TNFSF14 downregulation in AR.

CD83 is a well‐known marker of DC maturation and immune regulation [19]. Beyond its role as a surface marker, both membrane‐bound and soluble CD83 isoforms can modulate T and B cell responses [20]. Notably, soluble CD83 (sCD83) has immunosuppressive properties in allergy models: a recent study showed that administering sCD83 ameliorated experimental AR by altering antigen‐specific Th2 responses (reducing IL‐4/IL‐13) [21]. In our cohorts, CD83 expression was significantly lower in AR samples than in controls. The reduced CD83 expression observed in AR is intriguing given that CD83 is commonly considered a marker of dendritic‐cell activation. Several explanations may account for this observation, including differences in dendritic‐cell abundance, altered immune‐cell composition in bulk transcriptomic samples, disease‐stage‐specific regulation, or cohort‐related variation. Therefore, reduced CD83 expression should be interpreted as an immune‐associated transcriptional feature of AR rather than definitive evidence of impaired CD83‐mediated regulation. The concordance of our finding with prior work on sCD83 suggests that loss of CD83‐mediated regulation may be a feature of human AR. Overall, the dysregulated expression of TNFSF14 and CD83 in AR patients highlights their potential biological significance: TNFSF14's costimulatory signals and CD83's regulatory signals both appear altered, which may disrupt the balance of immune activation and tolerance in the nasal mucosa.

Our immune‐cell analyses provide further insight into how TNFSF14 and CD83 relate to AR pathophysiology. Using ssGSEA and CIBERSORT, we observed consistent alterations in T‐cell–related features in AR. Specifically, AR patients showed reduced enrichment of aDCs and Tfh cell signatures, alongside a relative increase in general T helper cell signatures. Similarly, CIBERSORT revealed a decrease in resting CD4+ memory T cells and an increase in naive CD4 + T cells in AR. These patterns are compatible with an environment of ongoing allergic inflammation. For instance, reduced Tfh cells (which normally help B cells produce high‐affinity IgG and IgA) may favor Th2‐dominant IgE responses [22, 23]. Conversely, elevated naive T cells and diminished memory T cells could indicate recruitment of memory cells to tissues or an influx of newly primed Th2 cells from the naïve pool. Our Pearson correlation analysis showed that both TNFSF14 and CD83 levels correlated with multiple T‐cell subsets. This suggests that fluctuations in these biomarkers may be tied to the overall T‐cell milieu in AR. For example, since CD83 is expressed on activated DCs and is essential for T cell priming [24], its downregulation could contribute to the skewed T‐cell homeostasis observed.

These findings align with the established immune cascade in AR: allergen exposure triggers mast cell degranulation and a Th2‐skewed cytokine milieu (notably IL‐4 and IL‐13) that drives late‐phase recruitment of eosinophils, basophils, and additional T cells into the nasal mucosa [25, 26, 27]. IL‐4/IL‐13 in particular enhance VCAM‐1 and other adhesion molecules on endothelium [28], perpetuating immune‐cell infiltration. Thus, our discovery of altered adhesion‐related genes and corresponding changes in immune‐cell profiles integrates well with known AR mechanisms. In summary, TNFSF14 and CD83 appear to mark key nodes in the AR immune network: TNFSF14 may influence T‐cell activation and survival, while CD83 reflects DC–T cell interactions that normally help resolve inflammation. Changes in these molecules may thereby contribute to the dysregulated type‐2 immune environment in AR.

Beyond biomarker identification, our analyses provide additional exploratory information regarding the regulatory context of TNFSF14 and CD83. We used NetworkAnalyst and miRTarBase to predict transcription factors and miRNA/lncRNA interactions upstream of TNFSF14 and CD83. Key predicted transcriptional regulators included members of the NF‐κB, STAT, and IRF families (Figure 3B), which are well known to control immune gene expression. For example, NF‐κB activation is a hallmark of allergen‐induced inflammation, and may directly regulate TNFSF14/CD83 transcription [29, 30]. The ceRNA network identified candidate microRNAs that might post‐transcriptionally modulate these genes; targeting such miRNAs could be a strategy to adjust TNFSF14 or CD83 levels. DSigDB‐based compound analysis identified several compounds associated with TNFSF14/CD83 expression signatures. However, these predictions should be interpreted cautiously because database‐derived associations do not necessarily indicate therapeutic efficacy or biological relevance in AR. Some identified compounds may reflect general transcriptional responses rather than AR‐specific regulatory effects. In summary, the TF and ceRNA analyses point to mechanistic links, and the drug predictions offer starting points for experimental testing of AR therapies.

Several limitations should be noted. First, this study is entirely based on secondary analysis of public gene expression data. While we validated findings across multiple datasets, experimental validation is needed. We did not measure protein levels or perform functional assays; thus, the biological effects of TNFSF14 and CD83 in AR remain to be tested in vitro or in vivo. Second, the sample sizes of the GEO cohorts were modest, particularly in the external validation datasets, which may limit statistical power and affect the stability of machine‐learning‐based feature selection. In small cohorts, algorithms such as LASSO, random forest, and SVM‐RFE may be sensitive to sample composition, potentially resulting in optimistic estimates of classifier performance and ROC metrics. Although the TNFSF14/CD83 model showed reproducible discrimination across independent datasets, the diagnostic performance, especially in cohorts with very limited sample sizes, should be interpreted cautiously and requires further validation in larger prospective cohorts. Relatedly, patient metadata (e.g., disease severity, treatment status) were sparse, preventing stratification by clinical factors. Although an inclusive DEG threshold was applied during the exploratory screening stage to capture potentially relevant candidates, this strategy may increase the possibility of false‐positive findings. Therefore, subsequent PPI network analysis and machine‐learning‐based prioritization were applied to further refine candidate genes. Third, our analysis focused on cell adhesion–related genes selected from curated databases, which may have missed other relevant AR biomarkers. Integrating additional omics data (e.g., proteomics, epigenetics) in future studies could uncover more markers. Fourth, bioinformatics predictions (TFs, ceRNAs, and compound associations) require cautious interpretation until experimentally confirmed, and the DSigDB‐based compound analysis should be considered hypothesis‐generating rather than evidence of therapeutic potential. In addition, AR often coexists with asthma or conjunctivitis; although all datasets labeled “AR,” the extent of comorbid disease was not controlled. Such heterogeneity could influence gene expression and immune cell profiles. Besides, because the analyzed cohorts represent specific populations and geographic backgrounds, the generalizability of the identified molecular patterns across different regions remains to be further investigated. Finally, because bulk transcriptomic datasets were analyzed, the observed expression changes may partly reflect alterations in immune‐cell composition rather than intrinsic regulation within specific cell populations.

Future work should address these limitations. We recommend validating TNFSF14 and CD83 expression by quantitative PCR and immunohistochemistry in new AR and control samples (nasal tissue and/or blood). Functional studies—such as modulating these genes in DC or T‐cell cultures, or using AR animal models—would test their causal role. The predicted upstream miRNAs and TFs could be examined by ChIP or reporter assays. It would also be valuable to correlate TNFSF14/CD83 levels with clinical parameters (symptom scores, IgE titers). Moreover, given the limited number of AR samples available for NMF clustering, the identified groups should be considered exploratory molecular patterns rather than definitive AR subtypes. Larger cohorts with independent validation are required to determine whether these patterns represent reproducible biological heterogeneity in AR. Regional and population‐specific differences may also contribute to variations in AR endotypes, as environmental exposure, genetic background, and immune characteristics may influence molecular profiles of allergic inflammation. For example, Berghi et al. reported associations between chemokine ligand 3 and allergic rhinitis in an Eastern European population, highlighting the potential contribution of population‐specific factors to AR‐related immune signatures [31]. In sum, our bioinformatic results generate numerous hypotheses for bench‐side and clinical research to pursue.

## 5 Conclusion

In conclusion, we have identified TNFSF14 and CD83 as candidate biomarkers identified from an adhesion‐associated immune landscape that distinguish AR patients from controls. These genes exhibited robust diagnostic performance and were associated with characteristic alterations in T‐cell–mediated immunity. Our data further reveal two molecular subtypes of AR with different immune and metabolic signatures, highlighting disease heterogeneity. Together, these findings suggest that TNFSF14 and CD83 not only serve as promising biomarkers but also advance our understanding of AR pathogenesis. Future validation of these targets could facilitate molecularly guided diagnosis and personalized therapy for AR.

## Author Contributions

**Guoshun He:** conceptualization, data curation, formal analysis, writing – review and editing, methodology, investigation, software, writing – original draft, resources. **Liying Zhang:** data curation, methodology, investigation, validation, writing – review and editing, visualization, project administration.

## Funding

The authors have nothing to report.

## Ethics Statement

The authors have nothing to report. This study doesn't include any human or animal testers.

## Conflicts of Interest

The authors declare no conflicts of interest.

## Supporting information


**Table S1:** Cell adhesion molecule‐related genes.
**Table S2:** MCODE Recognition Function Module.
**Table S3:** DSigDB database prediction results.

## Data Availability

All data analyzed in this work were downloaded from the Gene Expression Omnibus (GEO) public databases, including GSE75011, GSE46171 and GSE40037. Relevant database accession information has been provided in the manuscript.

## References

[1] L. Hossenbaccus , S. Linton , S. Garvey , and A. K. Ellis , “Towards Definitive Management of Allergic Rhinitis: Best Use of New and Established Therapies,” Allergy, Asthma & Clinical Immunology 16 (2020): 39, 10.1186/s13223-020-00436-y. PMC7251701 32508939

[2] C. D. Schwindt and R. Settipane , “Editorial,” supplement, American Journal of Rhinology & Allergy 26, no. S1 (2012): S1, 10.2500/ajra.2012.26.3844. 29025458

[3] C. F. Schuler Iv and J. M. Montejo , “Allergic Rhinitis in Children and Adolescents,” Immunology and Allergy Clinics of North America 41, no. 4 (2021): 613–625, 10.1016/j.iac.2021.07.010. 34602232

[4] H. A. Kakli and T. D. Riley , “Allergic Rhinitis,” Primary Care: Clinics in Office Practice 43, no. 3 (2016): 465–475, 10.1016/j.pop.2016.04.009. 27545735

[5] G. W. Canonica , J. Mullol , A. Pradalier , and A. Didier , “Patient Perceptions of Allergic Rhinitis and Quality of Life,” World Allergy Organization Journal 1, no. 9 (2008): 138–144, 10.1097/WOX.0b013e3181865faf. PMC3650970 23282577

[6] X. Zhang , M. Zhang , H. Sui , et al., “Prevalence and Risk Factors of Allergic Rhinitis Among Chinese Adults: A Nationwide Representative Cross‐Sectional Study,” World Allergy Organization Journal 16, no. 3 (2023): 100744, 10.1016/j.waojou.2023.100744. PMC10050653 37008533

[7] S. M. Nur Husna , H. T. Tan , N. Md Shukri , N. S. Mohd Ashari , and K. K. Wong , “Allergic Rhinitis: A Clinical and Pathophysiological Overview,” Front Med (Lausanne) 9 (2022): 874114, 10.3389/fmed.2022.874114. PMC9021509 35463011

[8] S. Akhouri and S. A. House , “Allergic Rhinitis. StatPearls. Treasure Island (FL) Ineligible Companies.” Disclosure: Steven House Declares No Relevant Financial Relationships With Ineligible Companies.: StatPearls Publishing Copyright © 2026 (StatPearls Publishing LLC, 2026).

[9] J. Bousquet , J. M. Anto , C. Bachert , et al., “Allergic Rhinitis,” Nature Reviews Disease Primers 6, no. 1 (2020): 95, 10.1038/s41572-020-00227-0. 33273461

[10] Y. He , Y. Chen , S. Xu , Y. Luo , F. Qin , and W. Hu , “Pathogenesis and Key Cells in Allergic Rhinitis,” International Archives of Allergy and Immunology 186, no. 5 (2025): 418–429, 10.1159/000541666. 39561729

[11] V. Singh , R. Kaur , P. Kumari , C. Pasricha , and R. Singh , “ICAM‐1 and VCAM‐1: Gatekeepers in Various Inflammatory and Cardiovascular Disorders,” Clinica Chimica Acta 548 (2023): 117487, 10.1016/j.cca.2023.117487. 37442359

[12] I. Ogulur , Y. Mitamura , D. Yazici , et al., “Type 2 Immunity in Allergic Diseases,” Cellular & Molecular Immunology 22, no. 3 (2025): 211–242, 10.1038/s41423-025-01261-2. PMC11868591 39962262

[13] M. Yang , L. Sun , D. Zhu , C. Meng , and J. Sha , “Recent Advances in Understanding the Effects of T Lymphocytes on Mucosal Barrier Function in Allergic Rhinitis,” Frontiers in Immunology 14 (2023): 1224129, 10.3389/fimmu.2023.1224129. PMC10523012 37771581

[14] W. Han , X. Pang , X. Yang , and L. Jiang , “Impacts of the Severity of Allergic Rhinitis on Inflammatory Characteristics, Nasal Function, Anxiety and Depression,” British Journal of Hospital Medicine 87, no. 1 (2026): 50388, 10.31083/BJHM50388. 41609148

[15] J. Xu , “The Role of Tumor Necrosis Factor Receptor Superfamily in Cancer: Insights Into Oncogenesis, Progression, and Therapeutic Strategies,” npj Precision Oncology 9, no. 1 (2025): 275, 10.1038/s41698-025-00990-x. PMC12328804 40770489

[16] R. S. Mousa , P. Invernizzi , J. L. Jones , and H. S. Mousa , “TNFSF14 (Light) in Intestinal Inflammation: Balancing Immune Activation and Resolution in IBD,” Frontiers in Immunology 16 (2025): 1657071, 10.3389/fimmu.2025.1657071. PMC12477249 41030453

[17] C. F. Ware , M. Croft , and G. A. Neil , “Realigning the LIGHT Signaling Network to Control Dysregulated Inflammation,” Journal of Experimental Medicine 219, no. 7 (2022): e20220236, 10.1084/jem.20220236. PMC9130030 35604387

[18] C. Quach , X. Li , P. Shafiei‐Jahani , et al., “BTLA Agonist Attenuates Th17‐driven Inflammation in a Mouse Model of Steroid‐Resistant Asthma,” Frontiers in Immunology 16 (2025): 1552394, 10.3389/fimmu.2025.1552394. PMC11986467 40226621

[19] A. Alshevskaya , S. Suleimanov , E. Sheveleva , et al., “Molecular Signatures of Dendritic Cell Activation Upon TNF Stimulation: A Multi‐Omics Study in Rheumatoid Arthritis,” International Journal of Molecular Sciences 26, no. 13 (2025): 6071, 10.3390/ijms26136071. PMC12250373 40649852

[20] B. Riaz , S. Islam , H. Ryu , and S. Sohn , “CD83 Regulates the Immune Responses in Inflammatory Disorders,” International Journal of Molecular Sciences 24, no. 3 (2023): 2831, 10.3390/ijms24032831. PMC9917562 36769151

[21] Y. J. Wu , Y. N. Song , X. R. Geng , et al., “Soluble CD83 Alleviates Experimental Allergic Rhinitis Through Modulating Antigen‐Specific Th2 Cell Property,” International Journal of Biological Sciences 16, no. 2 (2020): 216–227, 10.7150/ijbs.38722. PMC6949156 31929750

[22] M. Akiyama , W. Alshehri , S. Ishigaki , K. Saito , and Y. Kaneko , “Human T Follicular Helper Cells and Their Impact on IgE and IgG4 Production Across Allergy, Malignancy, and IgG4‐Related Disease,” Allergology International 74, no. 1 (2025): 25–32, 10.1016/j.alit.2024.07.005. 39164143

[23] P. F. Cañete and D. Yu , “Follicular T Cells and the Control of IgE Responses,” Allergology International 74, no. 1 (2025): 13–19, 10.1016/j.alit.2024.09.007. 39455298

[24] A. T. Prechtel , N. M. Turza , A. A. Theodoridis , and A. Steinkasserer , “CD83 Knockdown in Monocyte‐Derived Dendritic Cells by Small Interfering RNA Leads to a Diminished T Cell Stimulation,” Journal of Immunology 178, no. 9 (2007): 5454–5464, 10.4049/jimmunol.178.9.5454. 17442926

[25] N. J. Campion , S. Villazala‐Merino , R. S. Thwaites , et al., “Nasal IL‐13 Production Identifies Patients With Late‐Phase Allergic Responses,” Journal of Allergy and Clinical Immunology 152, no. 5 (2023): 1167–1178.e12, 10.1016/j.jaci.2023.06.026. 37536510

[26] N. T. Orban , M. R. Jacobson , K. T. Nouri‐Aria , S. R. Durham , and A. O. Eifan , “Repetitive Nasal Allergen Challenge in Allergic Rhinitis: Priming and Th2‐type Inflammation but No Evidence of Remodelling,” Clinical & Experimental Allergy 51, no. 2 (2021): 329–338, 10.1111/cea.13775. 33141493

[27] L. Tamasauskiene and B. Gradauskiene , “Profile of Immune Response During Nasal Challenge With Dermatophagoides Pteronyssinus in Subjects With Allergic Airway Diseases,” Journal of Inflammation 21, no. 1 (2024): 41, 10.1186/s12950-024-00415-9. PMC11526524 39482718

[28] K. Nakagome and M. Nagata , “The Possible Roles of IL‐4/IL‐13 in the Development of Eosinophil‐Predominant Severe Asthma,” Biomolecules 14, no. 5 (2024): 546, 10.3390/biom14050546. PMC11117569 38785953

[29] Y. Wang , X. Qi , H. Li , et al., “Inhibition of TRPV1 Attenuates Innate Nasal Epithelial Responses via NF‐κB Signaling Pathway in Allergic Rhinitis,” International Immunopharmacology 158 (2025): 114807, 10.1016/j.intimp.2025.114807. 40367690

[30] N. Arima , T. Kato , T. Kanno , D. Yamanaka , R. Tada , and Y. Adachi , “Mechanism of CD83 Expression Induction Through dectin‐1 and β‐glucan Interaction in Innate Immune Responses,” Immunology & Cell Biology 103, no. 9 (2025): 871–883, 10.1111/imcb.70053. 40820564

[31] O. Berghi , M. Dumitru , R. Caragheorgheopol , et al., “The Relationship Between Chemokine Ligand 3 and Allergic Rhinitis,” Cureus 12, no. 4 (2020): e7783, 10.7759/cureus.7783. PMC7243633 32461855

